# Supplementary figures and images for: Determining Minnesota bee species’ distributions and phenologies with the help of participatory science
Source: PeerJ. 2023 Nov 15;11:e16146. doi: 10.7717/peerj.16146 (PMC10656906; doi:10.7717/peerj.16146)

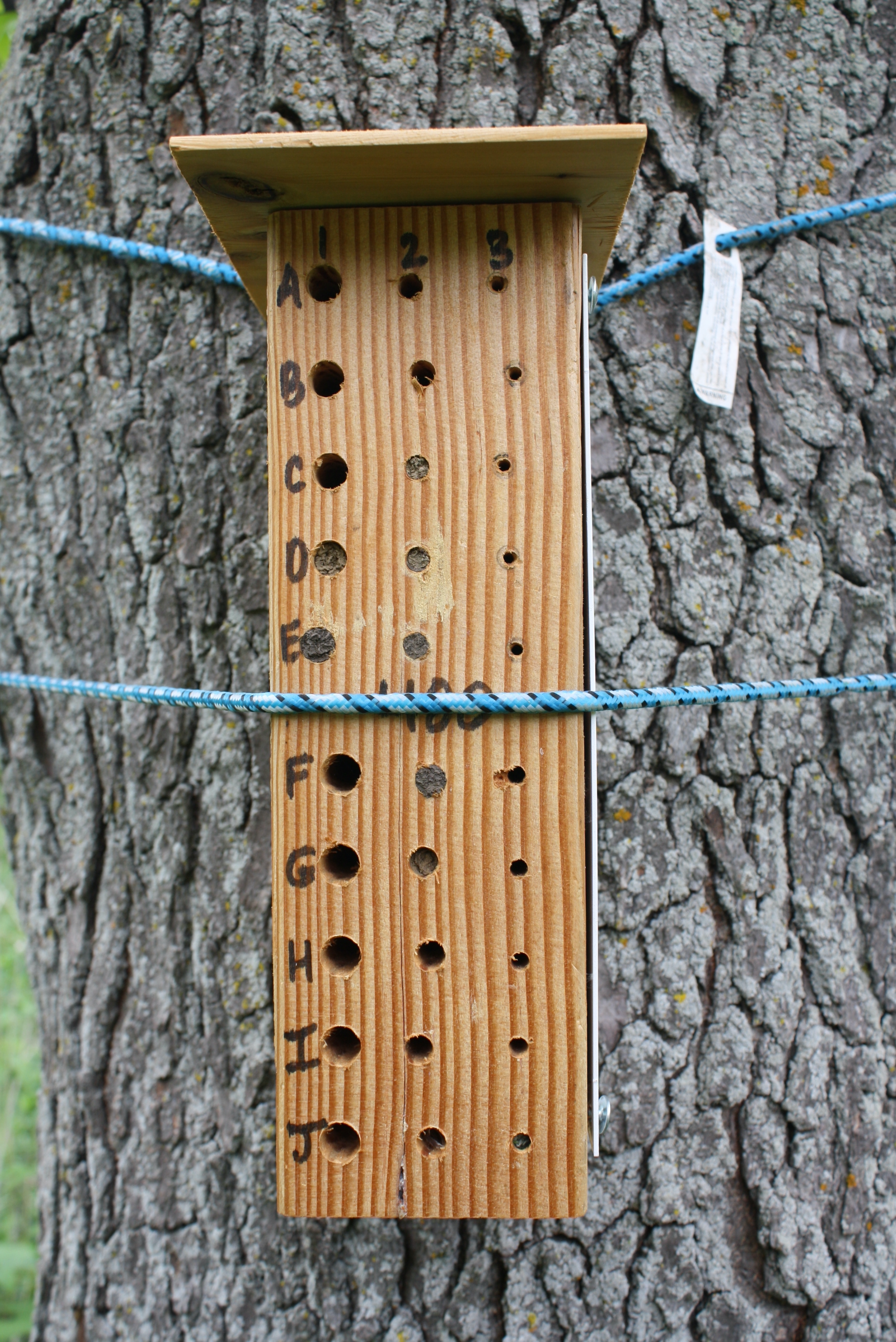

Supplement: Supplemental Information 1 — There are six different hole diameters: 3.18 mm, 4.76 mm, 6.35 mm, 7.94 mm, 9.53 mm, and 11.11 mm. [file peerj-11-16146-s001.png]
