# Supplementary material for: Determining Minnesota bee species’ distributions and phenologies with the help of participatory science
Source: PeerJ. 2023 Nov 15;11:e16146. doi: 10.7717/peerj.16146 (PMC10656906; doi:10.7717/peerj.16146)
Supplement: Supplemental Information 4 — Bumble bee species presence summed for all surveys along a route within a year. We removed the variable crops due to multicollinearity (variance inflation factor > 20), and the variable barren due to poor correlation (intra-set correlations with axes 1,2, or 3 < 0.4), and species accounting for less than 5% of the inertia for CCA 1 and 2 (Bombus rufocinctus Cresson, 1863 and B. vagans group). Significance of the overall CCA and ordination axes was determined with a Monte Carlo permutation test with 999 randomizations. [file peerj-11-16146-s004.docx]

Appendix 4.

In addition to the examination of the relationship of bumble bee species to land cover categories using correspondence analysis (CCA) using abundance data, we also used presence data for bumble bee species summed for all surveys along a route within a year. We removed the variable crops due to multicollinearity (variance inflation factor >20), and the variable barren due to poor correlation (intra-set correlations with axes 1,2, or 3 <0.4), and species accounting for less than 5% of the inertia for CCA 1 and 2 (*Bombus* *rufocinctus* Cresson, 1863 and *B.* *vagans* group). Significance of the overall CCA and ordination axes was determined with a Monte Carlo permutation test with 999 randomizations.

Bumble bee species abundance and land use were significantly correlated for the first two

canonical axes according to the Monte Carlo permutation test. Bumble bee species Axes CCA1

(eigenvalue=0.27, *F*=19.52, *p*<0.001) and CCA2 (eigenvalue=0.06, *F*=4.53, *p*<0.002) of the

correspondence analysis explained a cumulative 19% of the variation (Fig. 4A1). CCA1 primarily distinguished between grasslands and wetlands covers and CCA2 primarily distinguished between developed and grasslands covers (Table A4.1). Habitat associations for species with lower abundances may be due to chance (Legendre & Legendre, 2012), leading to caution interpreting habitat associations for these species due to their low abundances: *B.* *affinis* (17), *B.* *insularis* (2), *B. pensylvanicus* (22), and *Bombus* *flavidus* Eversmann, 1852 (36).

*Bombus* *fervidus* was associated with grassland land covers. *Bombus* *perplexus*, *B.* *insularis*, *B*. *terricola,* and possibly *B.* *flavidus* were associated with wetlands land cover. *Bombus ternarius* was associated with forested land cover. *Bombus* *auricomus* and possibly *B. pensylvanicus* and *B.* *affinis* were associated with developed land cover.

Table A4.1 **Biplot scores for constraining variables of land cover related to presence of bumble bee species.** The forest category combines deciduous, mixed, and evergreen forest. All levels of development were combined into the category. The grassland category includes grasslands/herbaceous and pasture/hay. The wetland category includes woody wetlands and emergent herbaceous wetlands. Correlations with absolute values ≥ 0.5 are bolded.

|  | CCA1 | CCA2 | CCA3 | CCA4 | CCA5 |
| --- | --- | --- | --- | --- | --- |
| developed | 0.466 | -0.859 | 0.201 | 0.057 | -0.033 |
| wetlands | -0.803 | -0.102 | -0.124 | 0.223 | 0.529 |
| forested | -0.759 | 0.034 | 0.186 | -0.616 | -0.095 |
| grasslands | 0.590 | 0.263 | 0.215 | -0.509 | 0.527 |
| open water | -0.102 | -0.220 | -0.829 | -0.502 | -0.048 |


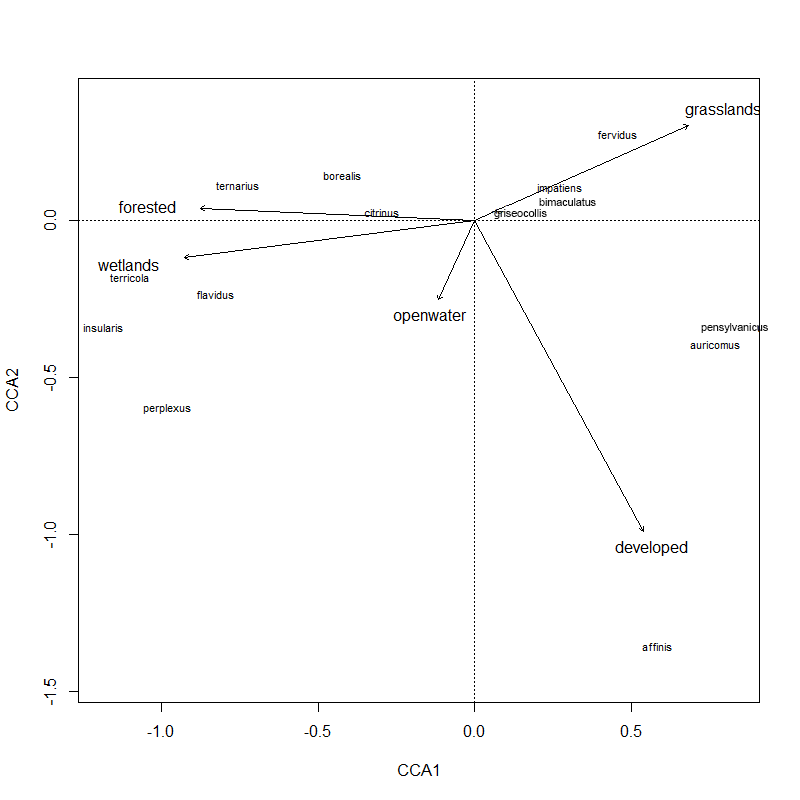


Figure A4.1 **Ordination showing the relationship of land cover to bumble bee species presence.** The location of each point relative to the arrows indicates the land cover variable associated with that species (Palmer 1993). Arrow length indicates the importance of the habitat variable in predicting the variability in the model (ter Braak 1986). Arrow direction indicates the strength of correlation with the axes with a small angle between arrow and axis indicating high correlation. Constrained correspondence analysis (CCA) axes 1 and 2 show the relationship of bumble bee species to land cover within 2 km of survey locations.
